# Wall Shear Stress Topological Skeleton Independently Predicts Long-Term Restenosis After Carotid Bifurcation Endarterectomy

**DOI:** 10.1007/s10439-020-02607-9

**Published:** 2020-09-14

**Authors:** Umberto Morbiducci, Valentina Mazzi, Maurizio Domanin, Giuseppe De Nisco, Christian Vergara, David A. Steinman, Diego Gallo

**Affiliations:** 1 PolitoBIOMed Lab, Department of Mechanical and Aerospace Engineering, Politecnico di Torino, Corso Duca degli Abruzzi, 24, 10129 Turin, Italy; 2 Department of Clinical Sciences and Community Health, Università di Milano, Milan, Italy; 3 Unità Operativa di Chirurgia Vascolare, Fondazione I.R.C.C.S. Cà Granda Ospedale Maggiore Policlinico, Milan, Italy; 4 Laboratory of Biological Structure Mechanics (LaBS), Dipartimento di Chimica, Materiali e Ingegneria Chimica ‘‘Giulio Natta’’, Politecnico di Milano, Milan, Italy; 5 Biomedical Simulation Laboratory, Department of Mechanical & Industrial Engineering, University of Toronto, Toronto, ON Canada

**Keywords:** Fixed points, Manifolds, Wall shear stress divergence, Computational fluid dynamics, Intima-media thickness

## Abstract

Wall Shear Stress (WSS) topological skeleton, composed by fixed points and the manifolds linking them, reflects the presence of blood flow features associated to adverse vascular response. However, the influence of WSS topological skeleton on vascular pathophysiology is still underexplored. This study aimed to identify direct associations between the WSS topological skeleton and markers of vascular disease from real-world clinical longitudinal data of long-term restenosis after carotid endarterectomy (CEA). Personalized computational hemodynamic simulations were performed on a cohort of 13 carotid models pre-CEA and at 1 month after CEA. At 60 months after CEA, intima-media thickness (IMT) was measured to detect long-term restenosis. The analysis of the WSS topological skeleton was carried out by applying a Eulerian method based on the WSS vector field divergence. To provide objective thresholds for WSS topological skeleton quantitative analysis, a computational hemodynamic dataset of 46 ostensibly healthy carotid bifurcation models was considered. CEA interventions did not completely restore physiological WSS topological skeleton features. Significant associations emerged between IMT at 60 months follow-up and the exposure to (1) high temporal variation of WSS contraction/expansion (*R*^2^ = 0.51, *p* < 0.05), and (2) high fixed point residence times, weighted by WSS contraction/expansion strength (*R*^2^ = 0.53, *p* < 0.05). These WSS topological skeleton features were statistically independent from the exposure to low WSS, a previously reported predictor of long-term restenosis, therefore representing different hemodynamic stimuli and potentially impacting differently the vascular response. This study confirms the direct association between WSS topological skeleton and markers of vascular disease, contributing to elucidate the mechanistic link between flow disturbances and clinical observations of vascular lesions.

**Electronic supplementary material:**

The online version of this article (10.1007/s10439-020-02607-9) contains supplementary material, which is available to authorized users.

## Introduction

Extensive research has investigated the mechanisms through which the hemodynamic environment at the carotid artery bifurcation influences the origin and progression of cardiovascular diseases.^27^ In particular, Wall Shear Stress (WSS) has been recognized as atherogenic,^27^ with previous evidence demonstrating that exposure to low^15^ and oscillatory^20^ WSS is a significant independent risk factor for identifying individuals at greater susceptibility for carotid atherosclerosis. The exposure to low WSS appears promising also in terms of predicting the risk of long-term restenosis after carotid endarterectomy (CEA), a surgical intervention consisting in the removal of the plaque on both symptomatic and asymptomatic patients with moderate to severe carotid stenosis.^28^ Long-term restenosis, an important complication affecting CEA outcome leading to development of cerebral symptoms or even carotid occlusion and stroke, presents similarities with native carotid artery stenosis^19^,^28^ when the absence of post-CEA residual atherosclerosis and short-term restenosis (i.e., > 6 months and < 24 months^14^,^19^) are accounted for. Thus, the mechanisms underlying both atherosclerosis and long-term restenosis are mechanistically influenced by the hemodynamic environment. However, the specificity of the currently considered hemodynamic features based on low and oscillatory WSS, which are significant but only moderate predictors of disease localization,^15^,^29^ and their clinical added value, hampered by the practical challenges of including hemodynamic information from computational modelling in large prospective clinical studies,^31^ have been questioned.^15^,^29^

To improve, refine and extend our current understanding of the association between local hemodynamics and vascular disease, an increasing interest has been recently dedicated to the analysis of WSS vector field topological skeleton,^5^,^6^,^25^ composed by fixed points and the stable/unstable manifolds connecting them. At a fixed point, the WSS vector field focally vanishes, and manifolds identify regions where the WSS vector field exerts a contraction/expansion action on the endothelial cells lining the luminal surface.^25^ It can therefore be presumed that WSS topological skeleton features influence vascular pathophysiology. This presumption is supported by the fact that the WSS topological skeleton is determined by blood flow structures associated to adverse vascular response at the carotid bifurcation,^25^ including near-wall flow stagnation, separation and recirculation.^6^ Moreover, the fluid-phase mass transport of solutes near the wall has been demonstrated to be governed by the cycle-average WSS topological skeleton.^3^,^6^,^13^ However, the exact mechanisms by which the WSS topological skeleton and related descriptors influence vascular pathophysiology are still underexplored.

The present study investigates the association between the WSS topological skeleton and markers of vascular disease at the carotid bifurcation from real-world, longitudinal clinical data. To do that, a cohort of 12 asymptomatic patients submitted to 13 CEA interventions^11^ was adopted. A recently proposed Eulerian-based analysis^25^ of the topological skeleton of the WSS vector field was applied to patient-specific computational hemodynamic models of the carotid bifurcation at 1 month after CEA. Intima-media thickness (IMT) was clinically measured at 60 months after CEA to provide an indicator of vascular response and detect the presence of long-term restenosis. Additionally, to explore how the CEA intervention impacts local hemodynamics and, ultimately, the clinical outcome, the WSS topological skeleton analysis was carried out on the pre-CEA (i.e., stenotic) carotid bifurcation models. For the purpose of contextualization of the results, the WSS topological skeleton analysis was (1) extended to a computational hemodynamics dataset of 46 ostensibly healthy carotid bifurcation models, and (2) complemented with the analysis of the exposure to low WSS, which was previously demonstrated to be directly associated to adverse vascular responses on the same post-CEA dataset adopted here.^11^

## Methods

### Patient Population Data

Endarterectomy procedures were performed on 13 carotid arteries in 12 patients at the Vascular Surgery Operative Unit of Fondazione IRCCS Ca’ Granda, Ospedale Maggiore Policlinico in Milan. All of the 13 carotid arteries had diameter stenosis of greater than 70%. As detailed elsewhere,^9^,^10^ all cases were asymptomatic, one case had contralateral occlusion of the internal carotid artery (ICA), and three cases were previously submitted to contralateral CEA. Age, sex, location of carotid stenosis, diameters of ICA and risk factors are listed in Table 1. The study was approved by the I.R.C.C.S. Fondazione Policlinico Ethics Committee according to institutional ethics guidelines, and participants provided informed consent.

**Table 1:** Patient data.

Patient	Age (years)	Sex	Clinical risk factors	Stenosis location	ICA Φ (mm)
PG1*	65	F	HTN	CCA, CB	5.00
PG2*	65	F	HTN	CB	5.40
PG3	81	F	HTN, SMOKE	CB, ICA	4.20
PG4	82	F	HTN	CB, ICA	4.00
PG5	72	M	HTN, DIAB, SMOKE	ICA	4.50
PG6	68	F	HTN, SMOKE	CB	4.50
PG7	71	F	HTN	CB, ICA	4.90
PG8	76	M	HTN, SMOKE	CB	4.00
PG9	67	M	HTN, DIAB, DYSLIP, SMOKE	CB, ICA	4.80
PC1	81	F	HTN, DYSLIP, SMOKE	CB, ICA	4.74
PC2	79	M	DIAB, DYSLIP	CB, ICA	5.00
PC3	79	M	HTN, DIAB, DYSLIP, SMOKE	CCA, CB, ICA	7.00
PC4	61	M	HTN, DYSLIP	CB	6.60

Age, classification of sex (F female; M male), clinical risk factors (HTN: presence of hypertension, DIAB: diabetes, DYSLIP: dyslipidemia, SMOKE: smoking), location of the carotid stenosis (CCA Common carotid artery; CB Carotid bulb; ICA Internal carotid artery; ECA external carotid artery), and diameter measurement at ICA (ICA Φ)

*Cases PG1 and PG2: respectively, right and left carotid of the same patient

After endarterectomy, patch graft angioplasty was performed in 9 cases (PG1-9) using a polyester collagen-coated patch (Ultra-thin Intervascular®, Mahwah, NJ U.S.A), and 4 cases underwent primary closure, i.e. without patch graft (PC1-4). Further details about the surgical cohort are reported elsewhere.^11^,^18^

All patients were then submitted to Doppler ultrasound (DUS) follow-up at 3, 24 and 60 months. Cases of restenosis were defined by a peak systolic velocity (PSV) of > 130 cm/s as measured by DUS (an indicator of the presence of a diameter stenosis greater than 50%,^1^ according to the European Carotid Stenosis Trial standard). No sign of restenosis and no symptoms of cerebrovascular ischemia emerged in any patient from follow-ups at 3 and 24 months. During the follow-up period, one patient died for myocardial infarction (PG4), and one for pancreatic carcinoma (PG8). All eligible patients were submitted to DUS follow-up at 60 months. Intima-media thickness (IMT) was measured using a Philips iU22 ultrasound scanner with linear 8 MHz probe (Philips Ultrasound, Bothwell, U.S.A) and automatically extracted offline with the clinical software Qlab (Philips Ultrasound, Bothwell, U.S.A) at the following locations: ICA distal to the carotid bulb (CB); CB; distal end of the common carotid artery (CCA), i.e., the flow divider (FD); CCA at 1 cm and 2 cm from the distal end of the CCA (FD-1cm and FD-2cm, respectively). As previously proposed,^11^ the maximum value of IMT found in the bifurcation region was also considered.

### Computational Hemodynamics

#### Pre-CEA and Post-CEA Cohorts

Magnetic resonance angiography (MRA) acquisitions were performed before and within a month after surgery to obtain the pre-CEA and post-CEA 3D geometry of the carotid bifurcations with a level set approach using the Vascular Modeling Toolkit software (VMTK, www.vmtk.org), as detailed elsewhere.^11^,^18^

Blood was modelled as an incompressible homogeneous Newtonian fluid,^23^,^26^ under laminar flow and rigid wall assumptions.^10^,^18^ The governing equations of fluid motion were solved numerically using the finite-element library LifeV (http://www.lifev.org) in discretized fluid domains discretized with tetrahedral meshes.^18^ Patient-specific flow rate waveforms were extracted at the CCA and ICA before and after CEA from echo-color DUS and imposed as boundary conditions in the numerical simulations. At the external carotid artery (ECA) outlet section, a traction-free condition was imposed. Details on image acquisition, mesh refinement study and computational settings are extensively described elsewhere.^10^,^11^,^18^

#### Healthy Cohort

To characterize the WSS topological skeleton features of the physiological carotid artery hemodynamics, and provide objective thresholds for quantitative analysis of the WSS topological skeleton descriptors introduced in the following, the topological skeleton analysis was performed on a previously characterized computational hemodynamics dataset of 46 ostensibly healthy carotid bifurcation models,^15^,^16^ denoted with the prefix He (He1–He46). Briefly, the 3D geometry of 46 carotid bifurcations was reconstructed from contrast enhanced MRA^30^ with a level set approach using the VMTK software and the governing equations of fluid motion were solved numerically using a validated in-house finite element solver^15^ with the same assumptions as the CEA dataset. Patient-specific flow rate waveforms were extracted from cine phase contrast magnetic resonance acquisitions at the CCA and ICA and imposed as boundary conditions in the numerical simulations, while at the ECA outlet section a traction-free condition was imposed.^15^ Further information on image acquisition, mesh refinement study and computational hemodynamic simulation of the ostensibly healthy carotid bifurcations is detailed elsewhere.^15^,^16^,^30^

### WSS Topological Skeleton Analysis and Quantitative Description

**Figure 1:**
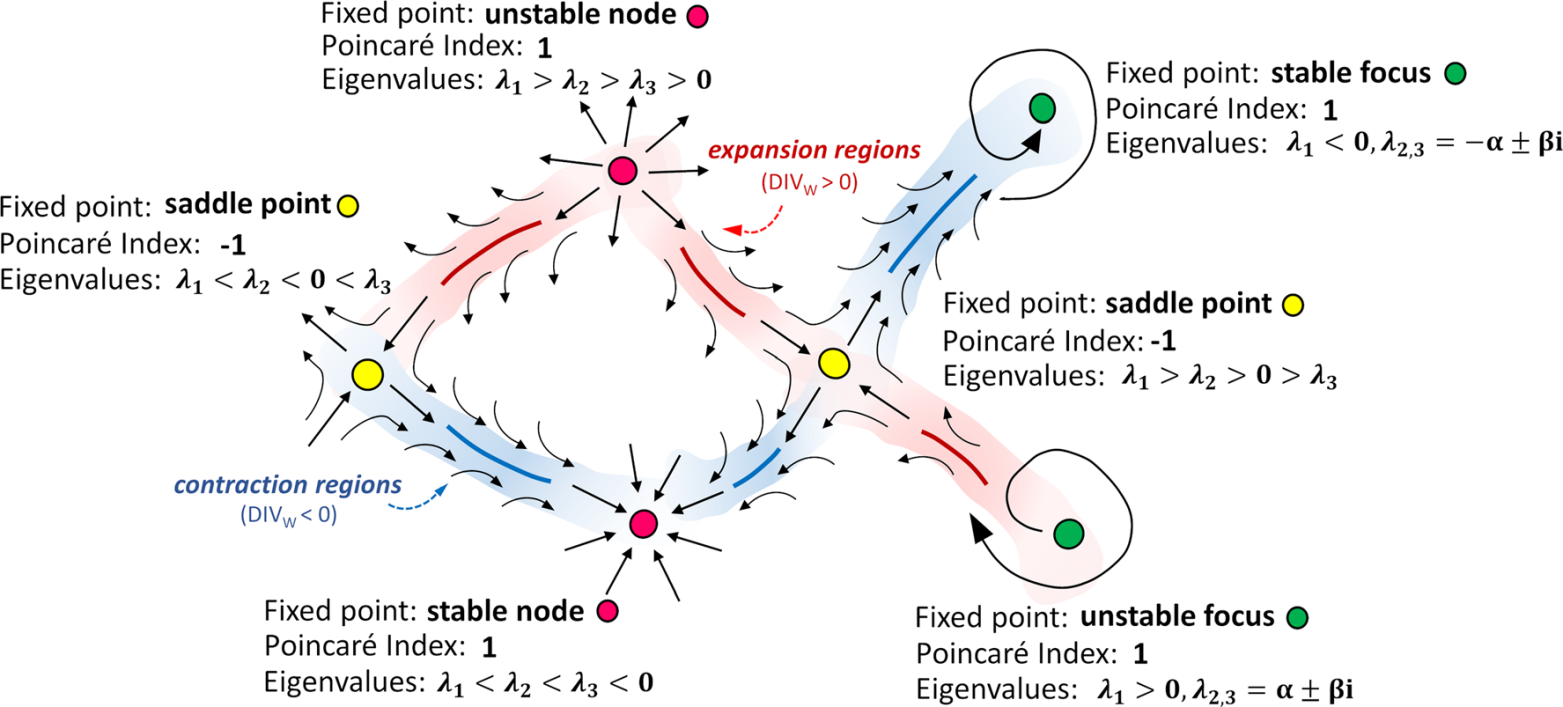
Explanatory sketch of the topological skeleton of a vector field. Configuration of each fixed point type and contraction/expansion regions, colored in blue/red respectively, are displayed. The Poincaré index (used for fixed points identification) and the eigenvalues of the Jacobian matrix (used for fixed points classification^25^) associated with each fixed point type are reported.

Starting from the WSS vector distribution at the luminal surface, the WSS topological skeleton analysis was carried out applying a recently proposed Eulerian method.^25^ Based on dynamical systems theory, the topological skeleton of the WSS field is composed by fixed points, i.e. points where the WSS vanishes, and manifolds, which identify WSS contraction/expansion regions (attracting/repelling manifolds, respectively) and connect the fixed points. An explanatory sketch of the WSS topological skeleton is presented in Fig. 1.

The method applied here rests on the Volume Contraction Theory and analyzes the WSS topological skeleton through the WSS vector field divergence and Poincaré index.^25^ In particular, it was demonstrated that the WSS manifolds, and hence the connections between WSS fixed points, can be encased by the divergence of the normalized WSS vector field^25^:

1 $${\text{DIV}}_{\text{W}} = \nabla \cdot({\varvec{\uptau}}_{u}) = \nabla \cdot \left( {\frac{{\varvec{\uptau}}}{{\left| {\varvec{\uptau}} \right|}}} \right),$$

where ${\varvec{\uptau}}$ is the WSS vector and ${\varvec{\uptau}}_{u}$ is its normalized (i.e., unit) version. The divergence of the normalized WSS vector field can be used to localize and identify the WSS spatial contraction/expansion configuration patterns at the carotid luminal surface with negative/positive values of DIV_W_, respectively (Fig. 1).^25^ To obtain the complete WSS topological skeleton, the WSS fixed points locations were determined using the Poincaré index.^25^ The possible Poincaré index values associated with each fixed point type (− 1 or + 1) are presented in Fig. 1. Then, the identified fixed points were classified according to their nature (saddle point, node or focus, Fig. 1), using the previously adopted approach based on the eigenvalues of the Jacobian matrix of the WSS vector field,^4^,^6^,^17^,^25^*λ*_1_, *λ*_2_, *λ*_3_ (with $\mathop \sum \limits_{i = 1}^{3} \lambda_{i} = \nabla \cdot\varvec{\uptau}$). As summarized in Fig. 1, three real eigenvalues with different signs identify a saddle point. Three real eigenvalues with the same sign identify a node, whose nature is characterized as attracting or repelling (i.e., stable or unstable, respectively)^17^ according to their sign (negative or positive, respectively, Fig. 1). Complex conjugate eigenvalues identify a stable or unstable focus, according to the sign of the real part (negative or positive, respectively, Fig. 1).

As a first step, the WSS topological skeleton of the cycle-average WSS vector field ${\bar{\mathbf{\uptau }}}$ at the luminal surface was analyzed. Subsequently, the WSS topological skeleton dynamics along the cardiac cycle was characterized. In order to measure the amount of variation in the WSS contraction/expansion action exerted at the carotid luminal surface along the cardiac cycle, here we adopted the quantity Topological Shear Variation Index (*TSVI*), defined as the root mean square deviation of the divergence of the normalized WSS with respect to its average over the cardiac cycle:

2 $${\textit{TSVI}} = \left\{ {\frac{1}{T}\mathop \smallint \limits_{0}^{T} \left[ { {\text{DIV}}_{\text{W}} - \overline{{{\text{DIV}}_{\text{W}} }} } \right]^{2} {\text{d}}t} \right\}^{1/2} = \left\{ {\frac{1}{T}\mathop \smallint \limits_{0}^{T} \left[ { \nabla \cdot\left( {{\varvec{\uptau}}_{u} } \right) - \overline{{\nabla \cdot({\varvec{\uptau}}_{u} )}} } \right]^{2} {\text{d}}t} \right\}^{1/2} ,$$

where *T* is the cardiac cycle duration and the overbar denotes a cycle-average quantity.

The unsteady nature of the WSS vector field fixed points along the cardiac cycle was characterized using the WSS fixed point weighted residence time along the cardiac cycle, as recently proposed:^25^

3 $$\it RT \nabla_{{x_{\textit{fp}} }} \left( e \right) = \frac{{A_{\text{avg}} }}{{A_{e} }}\frac{1}{T}\mathop \smallint \limits_{0}^{T} {\mathbb{I}}_{e} \left( {\varvec{x}_{\text{fp}} ,t} \right) | \left( \nabla \cdot\varvec{\uptau}\right)_{e} |\,{\text{d}}t,$$

where $\varvec{x}_{\textit{fp}} \left( t \right)$ is the location of a WSS fixed point at time $t \in \left[ {0,T} \right]$, *e* is the generic triangular element of the superficial mesh of area $A_{e}$ and $A_{\text{avg}}$ is the average surface area of all triangular elements of the superficial mesh of the luminal surface of the vessel, ${\mathbb{I}}_{e}$ is the indicator function and $(\nabla \cdot{\varvec{\uptau}})_{e}$ is the instantaneous WSS divergence value in* e*. The indicator function ${\mathbb{I}}_{e}$ equals 1 if $\varvec{x}_{\textit{fp}} \left( t \right) \in e$ (i.e., if the Poincaré index value is equal to − 1 or +1) and 0 otherwise (i.e., if the Poincaré index value is equal to 0). The quantity $RT\nabla_{{x_{\textit{fp}} }} \left( e \right)$ quantifies the residence time spent by a fixed point on the mesh element *e* of the carotid luminal surface. The residence time is expressed as fraction of the cardiac cycle and it is weighted by the strength of the local contraction/expansion action, given by the WSS divergence around the fixed point.^25^

In order to perform a quantitative analysis of the WSS topological skeleton, each pre-CEA, post-CEA and healthy carotid bifurcation was split in its CCA, ICA and ECA branches.^2^ The bifurcation region was delimited by sections located at 3, 5 and 2 radii along the CCA, ICA and ECA, respectively^22^ (denoted CCA3, ICA5 and ECA2). According to a previously employed threshold-based approach for the identification of regions at the luminal surface exposed to disturbed shear,^11^,^16^,^23^ here the exposure to large variations in the WSS contraction/expansion action was quantified by the relative surface area exposed to high values of *TSVI*, considering as threshold value the 80th percentile of the pooled *TSVI* distribution of the healthy models in the CCA3-ICA5-ECA2 region. This variable, denoted as Topological Shear Variation Area (TSVA), defines the relative area exposed to high normalized WSS divergence variability. Similarly, the exposure to the action of instantaneous WSS fixed points was quantified by the relative surface area exposed to non-null values of $RT\nabla_{{x_{\textit{fp}} }} \left( e \right)$, i.e. considering the luminal surface area where fixed points occurred along the cardiac cycle in the CCA3-ICA5-ECA2 region. This variable is denoted as weighted Fixed Points Area (wFPA).

To complement the WSS topological skeleton characterization, the luminal distribution of Time-Averaged Wall Shear Stress (*TAWSS*) magnitude along the cardiac cycle was also evaluated, as the exposure to low *TAWSS* values was previously linked to an increased long-term restenosis risk.^11^ The exposure to low *TAWSS* values was quantified by the relative surface area exposed to *TAWSS* values below a threshold value, corresponding to the 20th percentile of the pooled *TAWSS* distribution of the healthy models in the CCA3-ICA5-ECA2 region. This variable is denoted as Low Shear Area (LSA). Oscillatory WSS was not considered here as a previous investigation on the post-CEA cohort reported a not significant association with IMT.^11^

### Statistical Analysis

Differences among the three cohorts (i.e., pre-CEA, post-CEA and healthy) in terms of WSS features were evaluated with a Wilcoxon signed-rank test, with significance assumed for *p* < 0.05. The relationships between the relative exposure to high *TSVI*, $RT\nabla_{{x_{\textit{fp}} }} \left( e \right)$ and low *TAWSS* (respectively, TSVA, wFPA and LSA) were assessed with linear regression analysis. The quality of the regression was evaluated with the coefficient of determination *R*^2^. Significance was assumed for *p* < 0.05.

Successively, the nature of the relationship (if any) between WSS topological skeleton descriptors and clinical follow-up data was explored in the post-CEA dataset to test the physiological significance of WSS topological skeleton. Linear regression analysis was used to identify relationships between WSS topological skeleton descriptors with the measured IMT values. The quality of the regression was evaluated with the coefficient of determination *R*^2^. Significance was assumed for *p* < 0.05.

## Results

### Cycle-Average WSS Vector Field Topological Skeleton Analysis

**Figure 2:**
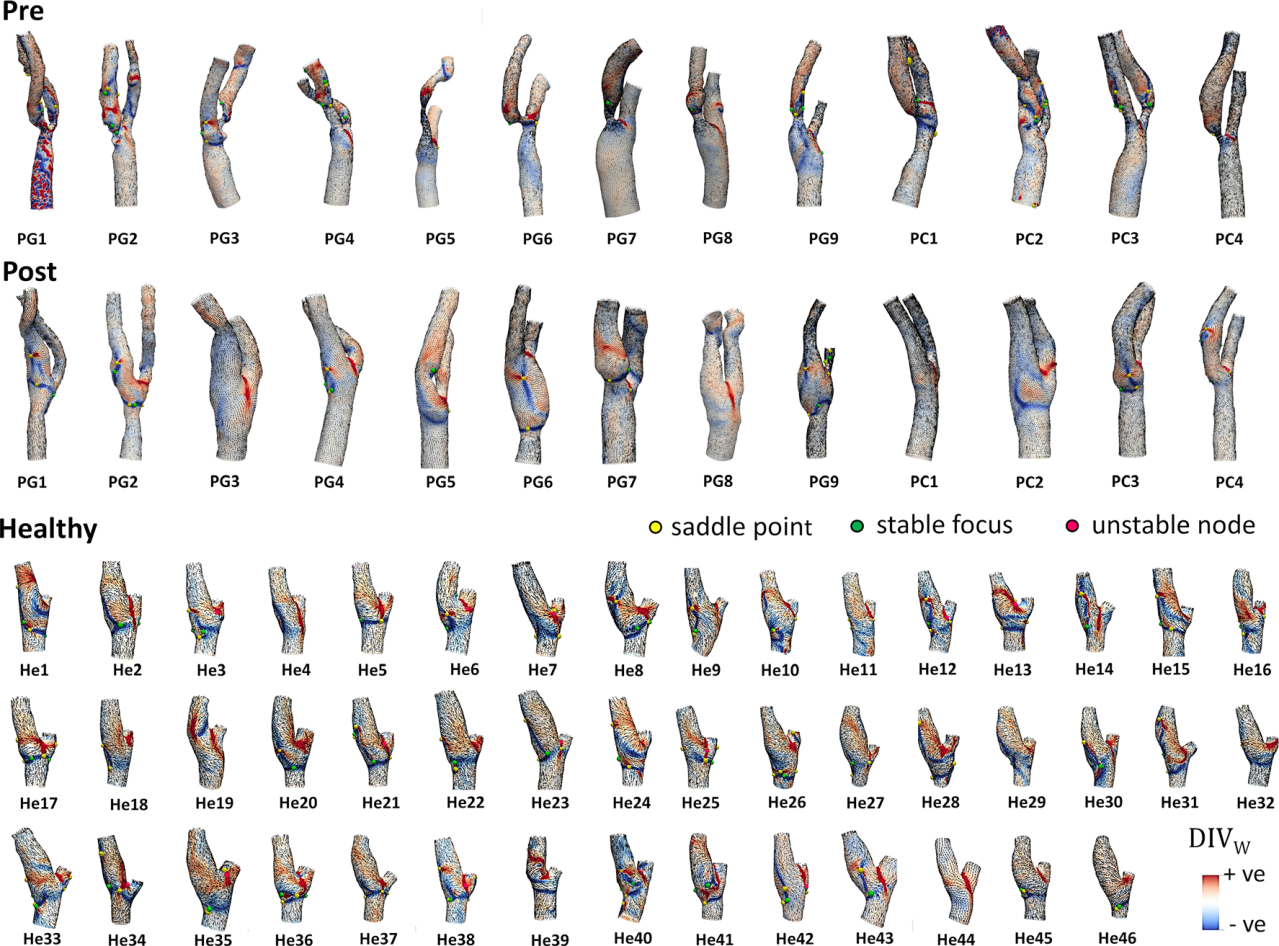
Topological skeleton of cycle-average WSS vector in pre-CEA (Pre), post-CEA (Post) and healthy cohorts. The topological skeleton in pre-CEA and post-CEA cohorts is extended beyond the bifurcation region (delimited by sections CCA3-ICA5-ECA2) to include in the pre-CEA models possible distal stenoses. Blue and red color define contraction and expansion regions, respectively, as given by the divergence of the normalized WSS vector field DIV_w_. The WSS vector field is normalized for visualization.

As a preliminary step, an exploration of the cycle-average WSS vector field topological skeleton was carried out to identify its main integral features on pre-CEA, post-CEA and healthy cohorts. Cycle-average WSS contraction/expansion regions, highlighted by the divergence of the normalized cycle-average WSS vector field, are presented in Fig. 2. In pre-CEA models, a WSS contraction region was located in correspondence of the cross-sectional area reduction at the stenosis, while for the post-CEA and healthy cohorts contraction and expansion regions were mainly located at the carotid bulb (Fig. 2), consistently with previous observations.^25^ A WSS expansion region was identified around the bifurcation apex as a feature common to all the carotid models (Fig. 2). As for the cycle-average WSS fixed points, saddle points, stable foci and unstable nodes were present on the carotid luminal surface of most of the carotid models, independent of the cohort, but at different locations: (1) in the pre-CEA cohort, cycle-average WSS fixed points were mostly located in proximity to the stenosis; (2) on post-CEA models, cycle-average WSS fixed points were located at the carotid bulb, in general with similarities to the cycle-average WSS topological skeleton of healthy carotid bifurcations (Fig. 2). A detailed analysis on the occurrence of cycle-average WSS fixed points for pre-CEA, post-CEA and healthy cohorts is reported in the Supplementary Material, highlighting that the healthy cohort presented on average the largest number of saddle points and unstable nodes in the bifurcation region (Fig. S1, Supplementary Material). Furthermore, the complete topological skeleton of the cycle-average WSS vector field can be better appreciated on selected representative cases for pre-CEA, post-CEA and healthy cohorts in Figure S2 of the Supplementary Material.

### WSS Topological Skeleton Dynamics Along the Cardiac Cycle

**Figure 3:**
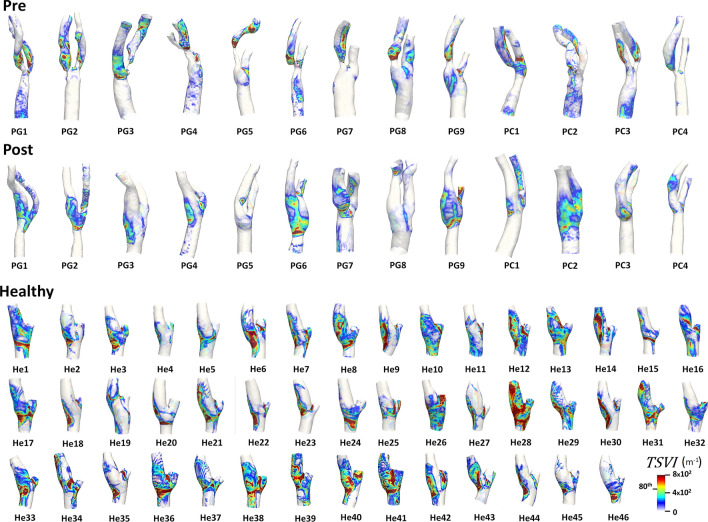
Luminal distribution of the Topological Shear Variation Index (*TSVI*) in pre-CEA (Pre), post-CEA (Post) and healthy cohorts. The *TSVI* distribution in pre-CEA and post-CEA cohorts is extended beyond the bifurcation region (delimited by sections CCA3-ICA5-ECA2) to include in the pre-CEA models possible distal stenoses. The 80^th^ percentile value of the pooled *TSVI* distribution of the healthy cohort in the bifurcation region is reported in the legend.

WSS topological skeleton was then analyzed along the cardiac cycle to account for its dynamics.^25^ The visualization of the *TSVI* luminal distributions in the pre-CEA and post-CEA cohorts (Fig. 3) was extended beyond the bifurcation region delimited by sections CCA3-ICA5-ECA2 to include in the pre-CEA models possible distal ICA/ECA stenoses. For the pre-CEA cohort, *TSVI* maps highlighted that the highest variations in the contraction/expansion action exerted by the WSS on the endothelium along the cardiac cycle were located mainly immediately downstream of the stenosis, where recirculating flow is expected. In the post-CEA cohort, high *TSVI* regions were observed at the cross-sectional enlargement in correspondence of the bifurcation, a known promoter of disturbed flow,^7^,^11^ and extended downstream in the ICA and ECA. Considering the *TSVI* luminal distributions in the healthy cohort (Fig. 3), the regions undergoing large variation in the WSS contraction/expansion action were in general located at the cross-sectional enlargement in the CCA, at the bulb in the ICA and around the bifurcation apex. For each investigated carotid model, the probability density function of *TSVI* is presented in the Supplementary Material (Figure S3), highlighting that low *TSVI* values are associated with the highest probability for all three cohorts. The extension of those high *TSVI* regions presented large interindividual variability (Fig. 3 and TSVA maps in Figure S4, Supplementary Material).

**Figure 4:**
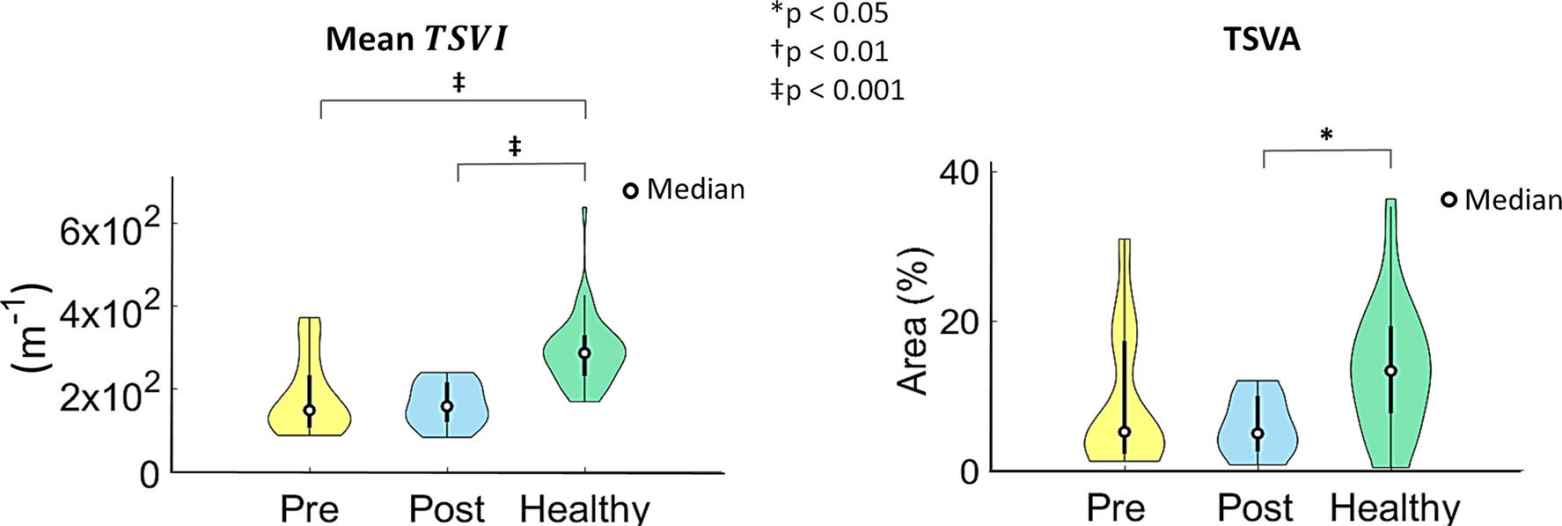
Violin plots of the mean value of the Topological Shear Variation Index (*TSVI*) and the Topological Shear Variation Area (TSVA) in the bifurcation region (delimited by sections CCA3-ICA5-ECA2) for pre-CEA (Pre), post-CEA (Post) and healthy cohorts. The distribution, median and quartile range are displayed for each cohort. Differences among the three cohorts are evaluated with a Wilcoxon signed-rank test.

In terms of *TSVI* averaged over the bifurcation region, marked differences in the distributions as well as significant differences emerged between the healthy and both pre- and post-CEA cohorts (*p* < 0.001), as highlighted by the violin plots in Fig. 4. To further characterize the high variations in the contraction/expansion action exerted by the WSS on the endothelium along the cardiac cycle, TSVA values were also evaluated in the three cohorts: (1) markedly different distributions were observed among the three cohorts (Fig. 4); (2) statistically significant differences emerged between post-CEA and healthy cohorts (*p* < 0.05, Fig. 4); (3) the post-CEA cohort exhibited the lowest intra-variability with respect to the other cohorts for both mean *TSVI* and TSVA (Fig. 4).

**Figure 5:**
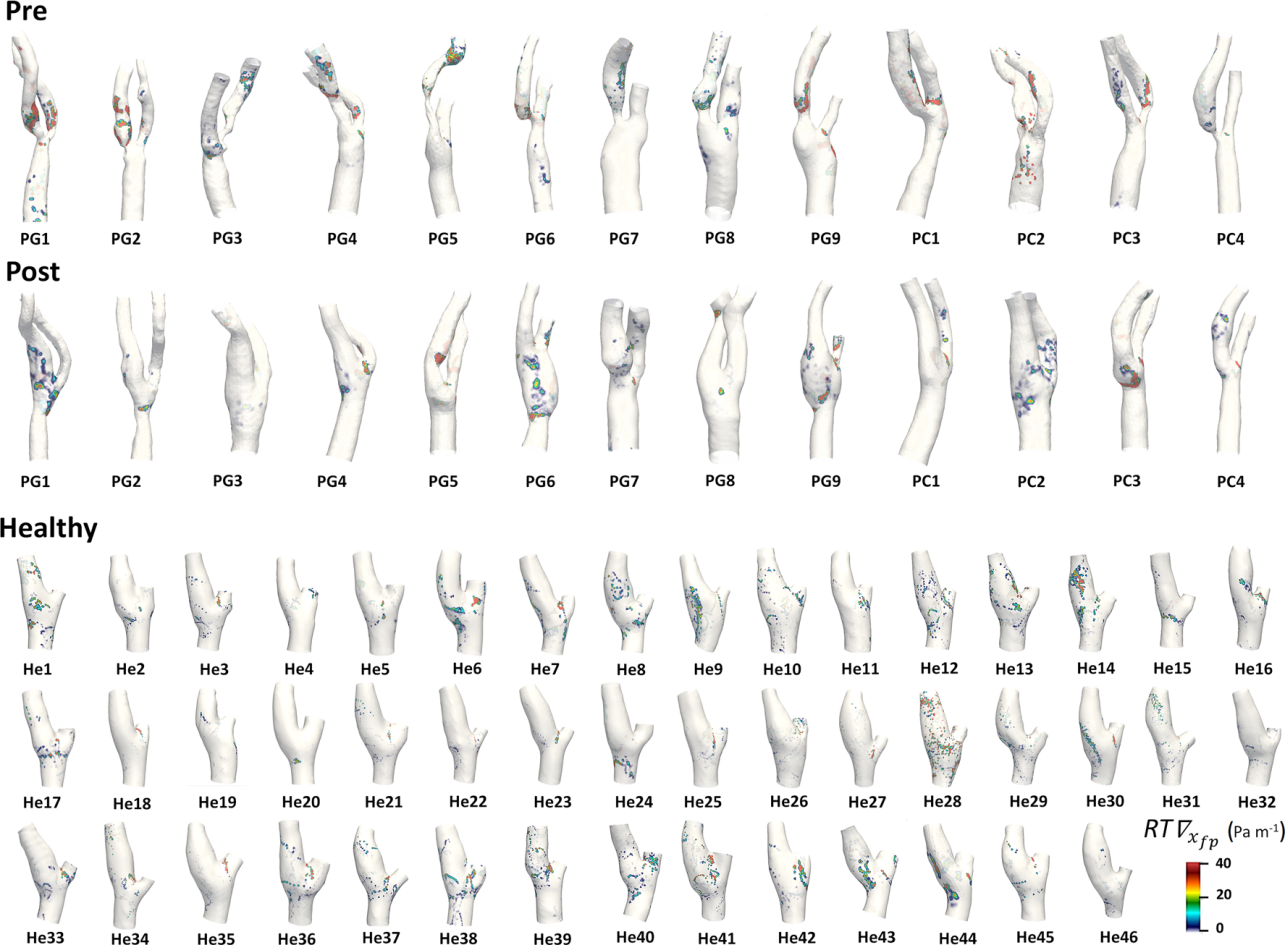
Luminal distribution of WSS fixed points weighted residence time ($RT\nabla_{{x_{\textit{fp}} }}$) in pre-CEA (Pre), post-CEA (Post) and healthy cohorts. The $RT\nabla_{{x_{\textit{fp}} }}$distribution in pre-CEA and post-CEA cohorts is extended beyond the bifurcation region (delimited by sections CCA3-ICA5-ECA2) to include in the pre-CEA models possible distal stenoses.

The analysis of the luminal surface distribution of WSS fixed points weighted residence time along the cardiac cycle highlighted their focal nature on the luminal surface of the carotid bifurcations, giving origin to a scattered distribution of non-null $RT\nabla_{{x_{\textit{fp}} }}$values (Fig. 5). In pre-CEA carotid models, the highest $RT\nabla_{{x_{\textit{fp}} }}$values were located immediately downstream of the stenosis, differing from the carotid models in the post-CEA and healthy cohorts, the latter exhibiting the lowest $RT\nabla_{{x_{\textit{fp}} }}$values. Moreover, a marked co-localization can be observed between high *TSVI* and high $RT\nabla_{{x_{\textit{fp}} }}$ regions at the luminal surface (Figs. 3 and 5, respectively).

**Figure 6:**
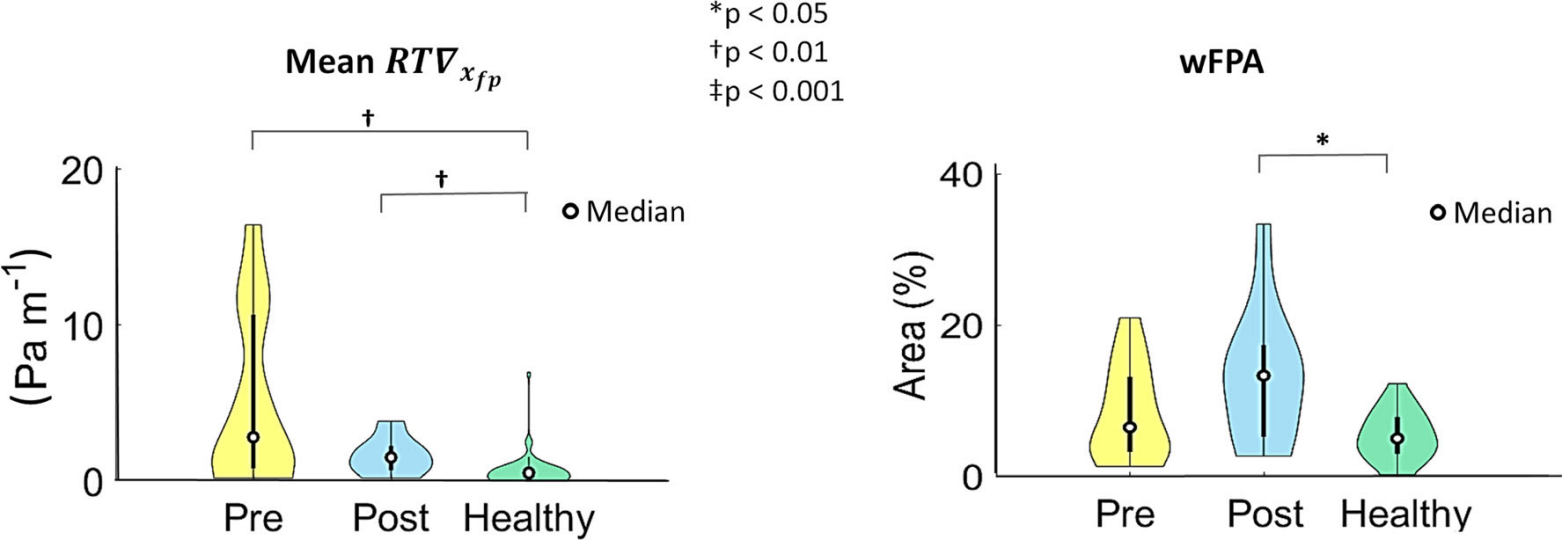
Violin plots of the mean value of WSS fixed points weighted residence time ($RT\nabla_{{x_{\textit{fp}} }}$) and weighted Fixed Points Area (wFPA) in the bifurcation region (delimited by sections CCA3-ICA5-ECA2) for pre-CEA (Pre), post-CEA (Post) and healthy cohorts. Distribution, median and quartile range are displayed for each cohort. Differences among the three cohorts are evaluated with a Wilcoxon signed-rank test.

Considering the values of $RT\nabla_{{x_{\textit{fp}} }}$ averaged over the bifurcation region, also in this case marked differences emerged in the distributions for the three cohorts (Fig. 6), as well as statistically significant differences between the healthy and both pre- and post-CEA cohorts (*p* < 0.01, Fig. 6). The exposure to high $RT\nabla_{{x_{\textit{fp}} }}$ values, quantified by wFPA, resulted significantly different between post-CEA and healthy cohorts only (*p* < 0.05, Fig. 6).

**Figure 7:**
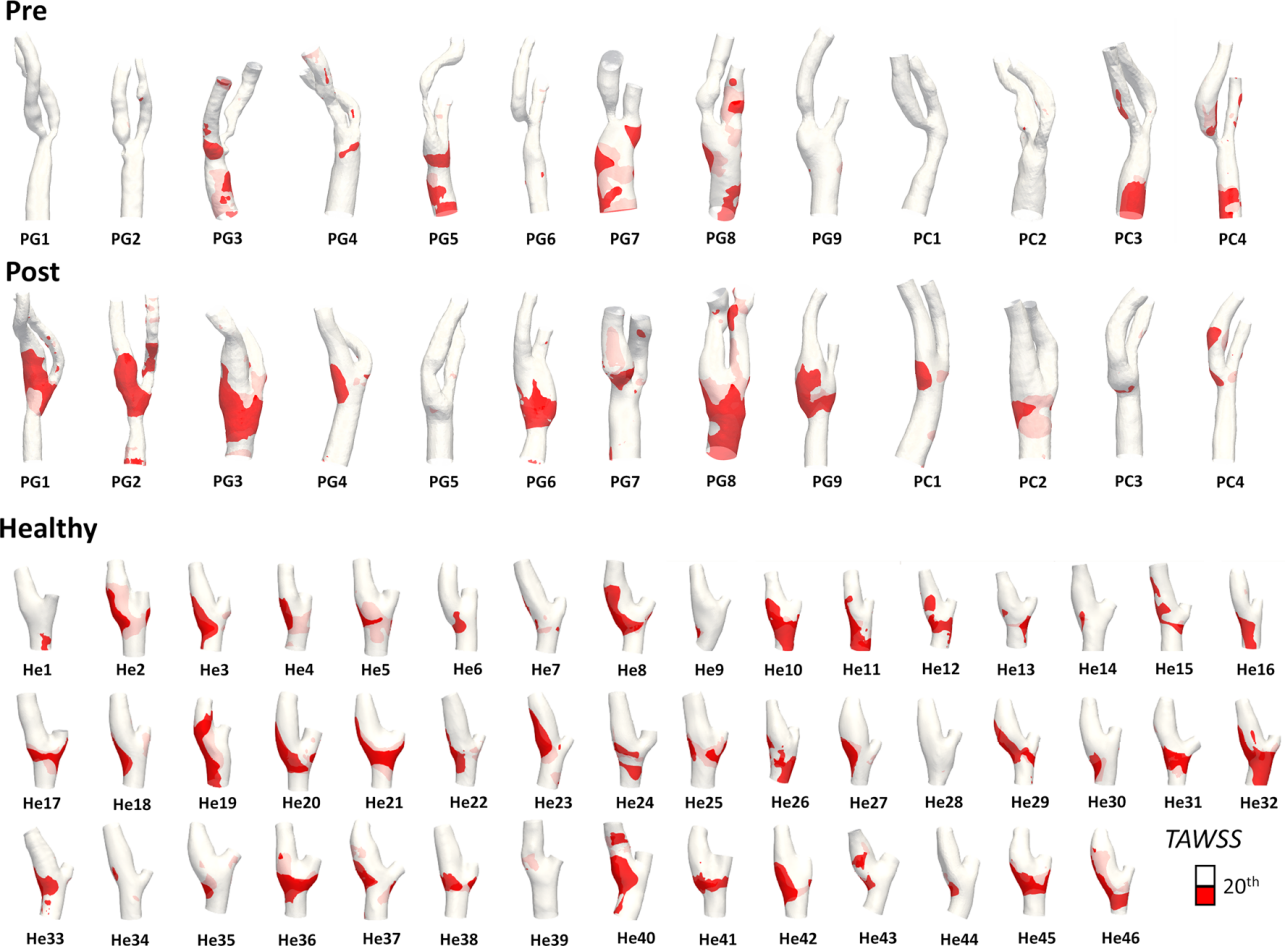
Luminal surface area exposed to low Time-Averaged Wall Shear Stress (*TAWSS*) as expressed by the Low Shear Area (LSA), in pre-CEA (Pre), post-CEA (Post) and healthy cohorts. The LSA in pre-CEA and post-CEA cohorts is extended beyond the bifurcation region (delimited by sections CCA3-ICA5-ECA2) to include in the pre-CEA models possible distal stenoses. Red areas represent *TAWSS* value below the 20th percentile of the pooled *TAWSS* distribution of the healthy models in the bifurcation region.

**Figure 8:**
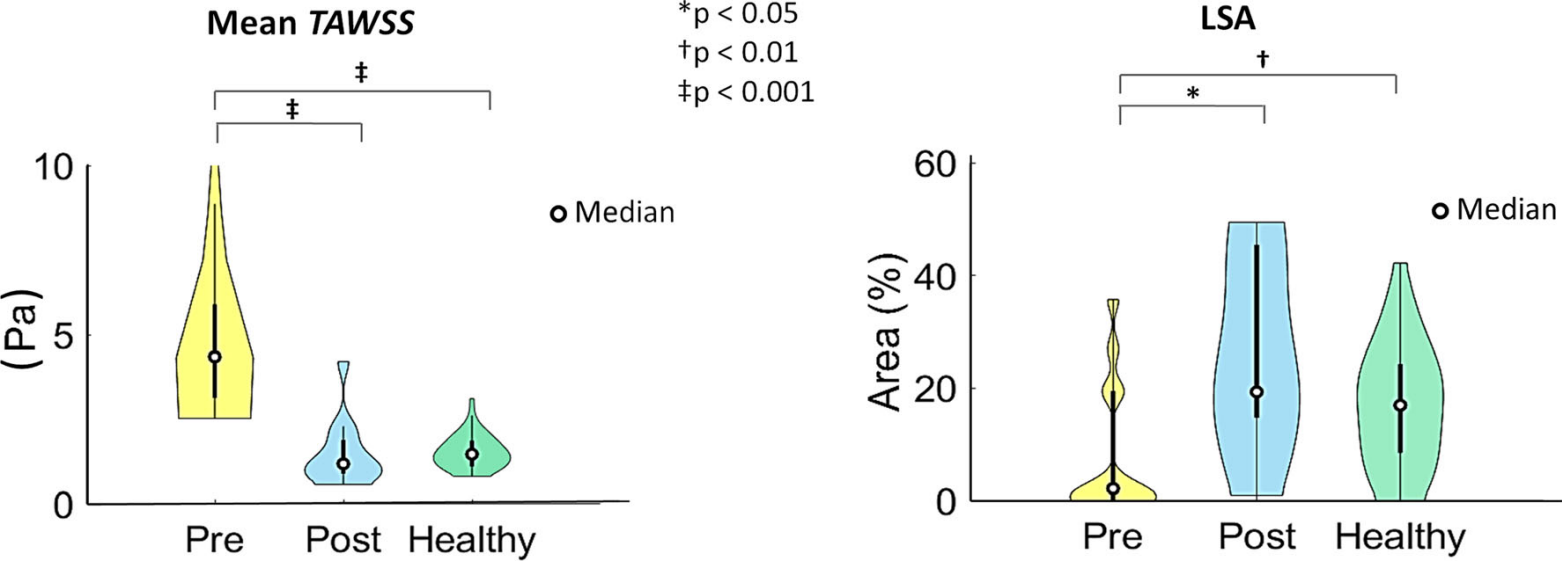
Violin plots of the mean value of Time-Averaged Wall Shear Stress (*TAWSS*) and Low Shear Area (LSA) in the bifurcation region (delimited by sections CCA3-ICA5-ECA2) for pre-CEA (Pre), post-CEA (Post) and healthy cohorts. Distribution, median and quartile range are displayed for each cohort. Differences among the three cohorts are evaluated with a Wilcoxon signed-rank test.

For each investigated carotid bifurcation model, the visualization of the surface area exposed to low *TAWSS* (LSA), highlighting wide interindividual variability, is reported in Fig. 7. The distribution of the values of *TAWSS* averaged over the bifurcation region in the three cohorts was markedly different between the pre-CEA and both post-CEA and healthy cohorts, as highlighted by the shape of violin plots in Fig. 8 and confirmed by the statistically significant differences between the healthy and both pre- and post-CEA cohorts (*p* < 0.01, Fig. 8). Statistically significant differences in LSA values in the bifurcation region emerged between the pre-CEA and both post-CEA and healthy cohorts (*p* < 0.05 and *p* < 0.01, respectively, Fig. 8), whereas the LSA in the bifurcation region for the post-CEA and healthy cohorts was not significantly different (Fig. 8).

### Relationships Among WSS Features

The coefficients of determination *R*^2^ between each couple of WSS-based descriptors are summarized in Table 2. As for the WSS topological skeleton, significant direct associations emerged between wFPA and TSVA for all three cohorts, ranging from *R*^2^ = 0.463 (*p* < 0.05) in the post-CEA cohort to *R*^2^ = 0.646 (*p* < 0.01) in the pre-CEA cohort (Table 2). For all three cohorts LSA was not associated to either wFPA or TSVA (Table 2), indicating that those WSS topological skeleton descriptors represent statistically independent variables with respect to the commonly adopted exposure to low *TAWSS* as a main indicator of disturbed shear in arteries.^11^,^15^,^27^

**Table 2:** Pairwise correlations among the relative exposure to high Topological Shear Variation Index expressed by the Topological Shear Variation Area (TSVA), non-null WSS fixed points weighted residence time expressed by the weighted Fixed Points Area (wFPA), and low Time-Averaged Wall Shear Stress expressed by the Low Shear Area (LSA).

Coefficient of determination *R*^2^	Pre		Post		Healthy	
	TSVA	LSA	TSVA	LSA	TSVA	LSA
wFPA	0.646‡	0.075	0.463‡	0.292	0.554‡	0.001
TSVA	–	0.193	–	0.164	–	0.011

The correlations are reported for the pre-CEA (Pre), post-CEA (Post) and healthy cohorts (Healthy)

‡*p* < 0.001

### Wall Shear Stress vs. Clinical Outcome

Linear regressions revealed significant associations between the WSS topological skeleton descriptors and IMT at 60 months follow up. In detail, a significant association emerged between maximum IMT and both TSVA (*R*^2^ = 0.505, *p* < 0.05) and wFPA (*R*^2^ = 0.534, *p* < 0.05), as reported in Table 3. A significant association was observed also between LSA and maximum IMT (*R*^2^ = 0.619, *p* < 0.001, Table 3). These associations, albeit slightly weaker (*p* < 0.05), were also observed considering the IMT values at the CB (Table 3). In the ICA distally to the CB, wFPA and LSA were significantly associated with the local IMT values (respectively, *R*^2^ = 0.541, *p* < 0.001 and *R*^2^ = 0.530, *p* < 0.05, Table 3), whereas TSVA was not. Scatter plots illustrating the significant correlations are reported in the Supplementary Material (Figure S5).

**Table 3:** Relationship between the hemodynamic variables Topological Shear Variation Area (TSVA), weighted Fixed Points Area (wFPA) or Low Shear Area (LSA) and intima-media thickness (IMT) measurements.

Coefficient of determination *R*^*2*^	TSVA	wFPA	LSA
Maximum IMT	0.505*	0.534*	0.619‡
IMT @ FD-2 cm	0.116	0.108	0.006
IMT @ FD-1 cm	0.004	0.271	0.046
IMT @ FD	0.032	0.161	0.272
IMT @ CB	0.474*	0.425*	0.421*
IMT @ ICA	0.090	0.541†	0.530*

Maximum IMT, IMT measured at the bifurcation level (flow divider FD), CCA at 2 cm and 1 cm proximal to the FD (FD-2 cm and FD-1 cm), at the CB, at the ICA downstream of the CB

CCA: common carotid artery, CB: carotid bulb, FD: flow divider, ICA: internal carotid artery

*p < 0.05; †*p* < 0.01; ‡*p* < 0.001

## Discussion

WSS topological skeleton features reflect cardiovascular flow complexity,^3^,^4^,^6^,^25^ with direct links to arterial flow patterns like near-wall flow stagnation, separation and recirculation, which are known to be promoting factors for cardiovascular disease.^6^,^27^ In this sense, the role of WSS topological skeleton in vascular pathophysiology is currently based on circumstantial evidence documenting how the complex flow features associated to the WSS topological skeleton induce a focal vascular response.^6^ At the carotid bifurcation, the extent of flow recirculation has been shown to correlate with atherosclerotic biomarkers,^24^ while flow stagnation and separation at the carotid bulb have been associated to endothelial dysfunction^15^ and intimal thickening,^32^ respectively. In addition, further circumstantial evidence about the role of the WSS topological skeleton in vascular disease has been provided by previous studies demonstrating that the cycle-average WSS topological skeleton governs the near-wall mass transport in arteries,^3^,^6^,^13^ a process linked to the onset and progression of early atherosclerosis.^12^ Here, we directly link the WSS topological skeleton to the vascular response after 60 months follow-up, defined by clinical IMT measurements. The IMT measurements additionally provide an indicator of restenosis, a common adverse event of CEA procedures.^28^ As a main finding of the study, we report that the investigated WSS topological descriptors TSVA and wFPA were associated with the IMT measurements at 60 months follow-up after CEA in the CB and in the ICA (*R*^2^ up to 0.541, *p* = 0.009 as presented in Table 3). Albeit the strength of these associations is moderate, it is comparable to the strength of the correlation between exposure to low WSS and IMT measurements at 60 months follow-up (Table 3).

Distilling these correlations into mechanistic implications, the here-proposed topological skeleton analysis suggests that exposure to (1) high temporal variation of WSS contraction/expansion action on the endothelium (quantified by TSVA) and (2) high residence times of fixed points at the luminal surface, weighted by WSS contraction/expansion strength (quantified by wFPA), may act as biomechanical triggers of long-term restenosis after CEA, a process anecdotally anticipated to vascular surgeons by the presence of flow disturbances.^28^ In other words, our findings support the hypothesis that the WSS topological skeleton features here considered could contribute to promote long-term restenosis, which represents recurrent atherosclerosis.^19^,^28^ This is corroborated by the fact that, in post-CEA cohort: (1) within 3 months of CEA no sign of lesions (which would represent residual atherosclerosis rather than restenosis^28^) was reported; (2) short-term restenosis, developing between 6 and 24 months postoperatively subsequently to neointimal hyperplasia,^28^ was not observed clinically after 24 months from CEA. Therefore, the approach presented here potentially contributes to a deeper understanding of the hemodynamics-driven processes underlying long-term restenosis development in the carotid bifurcation and could be extended to the study of biomechanical triggers of atherosclerosis and vascular disease. In this regard, we recently suggested a link between the variation of the WSS contraction/expansion action and wall stiffness in patients affected by ascending aortic aneurysm.^8^

To investigate more in depth the physiological significance of the WSS topological skeleton features, the analysis was extended to a dataset of ostensibly healthy carotid bifurcation models. By comparing the pre-CEA, post-CEA and healthy cohorts, it was possible to understand to what extent the pathological pre-CEA near-wall hemodynamics can be restored towards a more physiological condition as a result of the CEA intervention. Interestingly, on average it emerged that differences in WSS topological skeleton features with respect to the healthy carotid bifurcations persisted after the CEA intervention (Figs. 4 and 6). Moreover, the contribution of saddle points and foci to the wFPA was associated to maximum IMT, IMT values at the CB and at the ICA distally to the CB (*R*^2^ up to 0.557, *p* < 0.01 as reported in Table S1, Supplementary Material), while the contribution of nodes to the wFPA was weakly associated to IMT values measured at 2 cm from the distal end of the CCA (*R*^2^ = 0.390, *p* < 0.05, Table S1, Supplementary Material). This suggests an influence of the type of WSS fixed point on the associations between wFPA and IMT measurements at 60 months.

On the same post-CEA cohort adopted here, a significant direct association between the exposure to low WSS (quantified by LSA) with maximum IMT at 60 months follow up after CEA was previously reported.^11^ An exact understanding of the mechanistic process underlying the development of carotid restenosis after CEA has not yet been achieved; however, the present findings expand the current hypothesis that larger LSAs lead to an increased long-term restenosis risk,^11^ by demonstrating that other hemodynamic features besides low shear are independently linked to long-term restenosis. These features are obtained starting from the WSS topological skeleton and quantified by the WSS topological descriptors wFPA and TSVA. The statistical independence between both wFPA and TSVA and LSA in all three examined cohorts (reported in Table 2) suggests that these WSS topological skeleton features and low WSS represent different hemodynamic stimuli, possibly impacting differently the vascular response. Consistently, the co-localization of high *TSVI* and high $RT\nabla_{{x_{\textit{fp}} }}$ regions with low cycle-average WSS regions was moderate for the post-CEA and healthy cohorts, and poor for the pre-CEA cohort (Figs. 3, 5 and 7), where a severe stenosis might induce a marked flow recirculation characterized by large variations in the WSS contraction/expansion action, high fixed points residence time, but concurrently relatively high cycle-average WSS. As a consequence of these observations, in principle the prediction of the long-term restenosis risk by hemodynamic analysis might be improved by taking into account not only the amount of time-averaged low shear,^11^ but also the introduced descriptors based on WSS topological skeleton. As previously reported on the same post-CEA cohort adopted here,^11^ the exposure to oscillatory WSS was not associated to IMT, thereby suggesting differences in the vascular response to focal (i.e., point-based) WSS oscillatory directional changes with respect to directional changes in the neighborhood of a point leading to variations in the contraction/expansion action.

On the other hand, although the different physical meanings underpinning the two WSS topological skeleton descriptors wFPA and TSVA (i.e., exposure to non-null values of the residence time of a fixed point, weighted by the local WSS contraction/expansion action *vs*. exposure to high normalized WSS divergence variability, respectively), a significant association between them emerged in all three cohorts (Table 2). This was consistent with the observed co-localization between luminal surface areas exposed to high *TSVI* and $RT\nabla_{{x_{\textit{fp}} }}$ in all models (Figs. 3 and 5, respectively), with the former encompassing the latter. Consequently, fixed points occurred in regions where normalized WSS divergence variations were high (Fig. 3) and the contraction/expansion regions connecting fixed points were characterized by both high normalized WSS divergence absolute values and high normalized WSS divergence variations (Fig. 3).

At 60 months after CEA, restenosis occurred in post-CEA carotid models PG1 and PG2, with diameter stenosis > 70% and > 50% respectively.^11^ Notably, in the post-CEA cohort, PG1 was characterized by the highest wFPA value, while PG2 had the highest TSVA value (Figs. 3 and 5). Those two cases were also characterized by the highest LSA values in the post-CEA cohort, as can be seen in Fig. 7 and as previously reported,^11^ although using a different *TAWSS* threshold value to define LSA. A marked intima-media thickening was also observed^11^ at 60 months follow-up in post-CEA patients PG3 and PC2 at the FD, PG6 in the CCA (FD-2 cm), in correspondence of either low cycle-average WSS (PG3, Fig. 7) or large variations in the WSS contraction/expansion and weighted fixed point residence times (Figs. 3 and 5, respectively).

This study faces possible limitations. Among them, we mention differences between the CEA patients and healthy cohorts, mainly in terms of cohort size and mean age (72.8 ± 7.2 vs. 58.7 ± 11.8, respectively). These differences can be partially ascribed to the clinical real-world nature of the data adopted for the CEA cohorts, which however allowed to address the typical challenges related to longitudinal studies (e.g., long time-scale of the vascular pathophysiology processes, patients’ recruitment and follow-up). Moreover, randomization was not performed for the selection of the CEA patients, and the exact extension of the region that underwent CEA surgical intervention (either with or without graft) could not be extracted from the imaging data. The relationships here reported might be influenced by the uncertainties (e.g., reconstruction errors) and assumptions/idealizations (e.g., Newtonian viscosity, rigid walls, as widely discussed elsewhere^21^,^31^) affecting computational hemodynamics. Because of these limitations, future investigations are warranted to further confirm the validity of the relationships presented here.

In conclusion, our study confirms what has been inferred in previous studies^4^,^6^,^8^,^25^ on the WSS topological skeleton: WSS topological skeleton features are associated to markers of vascular disease. High variability in the WSS contraction/expansion and high WSS fixed points weighted residence times at 1 month after CEA are correlated to IMT measurements at 60 months follow-up as markers of vascular disease at the carotid bifurcation. Moreover, the findings of this study may help in clarifying the role played by hemodynamics in the mechanisms underlying the development of long-term carotid restenosis after CEA (and, by extension, of atherosclerosis), demonstrating that WSS topological skeleton features might represent a different hemodynamic cue with respect to low WSS. Nevertheless, further investigations detailing and elucidating the effects of the WSS topological skeleton on vascular pathophysiology are encouraged. In this regard, the here applied Eulerian-based method for topological skeleton analysis^25^ confirms its potential as an effective biomechanical tool for increasing the chance of elucidating the mechanistic link between flow disturbances and clinical observations.

## Electronic supplementary material

Below is the link to the electronic supplementary material.

Supplementary material 1 (PDF 811 kb)
