# Supplementary material for: Wall Shear Stress Topological Skeleton Independently Predicts Long-Term Restenosis After Carotid Bifurcation Endarterectomy
Source: Ann Biomed Eng. 2020 Sep 14;48(12):2936–49. doi: 10.1007/s10439-020-02607-9 (PMC7723943; doi:10.1007/s10439-020-02607-9)
Supplement: Supplementary file 1 — Supplementary material 1 (PDF 811 kb) [file 10439_2020_2607_MOESM1_ESM.pdf]

# - SUPPLEMENTARY MATERIAL -

WALL SHEAR STRESS TOPOLOGICAL SKELETON INDEPENDENTLY PREDICTS LONG-TERM RESTENOSIS AFTER CAROTID BIFURCATION ENDARTERECTOMY

Umberto Morbiducci [a] *, Valentina Mazzi [a] *, Maurizio Domanin [b-c], Giuseppe De Nisco [a], Christian Vergara [d], David A. Steinman[e], Diego Gallo [a ⱡ]

[a] Polito[BIO]Med Lab, Department of Mechanical and Aerospace Engineering, Politecnico di Torino, Turin, Italy

[b] Department of Clinical Sciences and Community Health, Università di Milano, Italy

[c] Unità Operativa di Chirurgia Vascolare, Fondazione I.R.C.C.S. Cà Granda Ospedale Maggiore Policlinico, Milano, Italy

[d] Laboratory of Biological Structure Mechanics (LaBS), Dipartimento di Chimica, Materiali e Ingegneria Chimica ''Giulio Natta'', Politecnico di Milano, Italy

[e] Biomedical Simulation Laboratory, Department of Mechanical & Industrial Engineering, University of Toronto, Toronto, ON Canada

[*] The authors contributed equally to this study

[ⱡ] Address for correspondence:

Diego Gallo, PhD

Polito[BIO]Med Lab, Department of Mechanical and Aerospace Engineering

Corso Duca degli Abruzzi, 24 – 10129 Turin, Italy

Email: diego.gallo@polito.it

SUPPLEMENTARY RESULTS

In violin plots of Figure S1, the distribution, median and quartile range are presented for the occurrence of cycle-average WSS fixed points in the bifurcation region for pre-CEA, post-CEA and healthy cohorts. In general, in the bifurcation region delimited by sections CCA3-ICA5-ECA2, the distribution of the different fixed points types in the three cohorts was markedly different, except for unstable nodes in pre- and post-CEA models (as highlighted by the shape of violin plots in Figure S1). More in detail, no cycle-average WSS fixed point was observed in two pre-CEA models (PG4 and PG8, Figure 2 in the main article and Figure S1) and in one healthy model (He4, Figure 2 in the main article and Figure S1), while all post-CEA models presented at least one cycle-average WSS fixed point. Focusing the analysis on the occurrence of saddle points, three pre-CEA models did not present any saddle point in the bifurcation region (PG4, PG7 and PG8, Figure 2 in the main article and Figure S1), while in the post-CEA cohort all models presented at least one saddle point. Considering the carotid bifurcations in the healthy cohort, on average they exhibited a higher number of saddle points in the bifurcation region than the other two cohorts (median number of saddle points in pre-CEA cohort: 1, post-CEA cohort: 3, healthy cohort: 4, Figure 2). Statistically significant differences emerged among the three cohorts in terms of occurrence of saddle points (Figure S1). Concerning the presence of cycle-average WSS stable foci in the bifurcation region, five pre-CEA models did not exhibit any (PG4, PG5, PG8, PC3 and PC4, Figure 2 in the main article and Figure S1). In consequence of the CEA, the number of stable foci in the bifurcation region increased or remained constant in eleven models, while it decreased to zero for two models (PC1 and PC2). Cycle-average WSS stable foci were observed in all but two healthy carotid models (He4 and He7). Statistically significant differences in the occurrence of stable foci emerged between the pre-CEA and healthy cohorts (p=0.026, Figure S1). Unstable nodes were identified in the bifurcation region of one pre-CEA model only (PC1, Figure 2 in the main article). After CEA, cycle-average WSS unstable nodes appeared in models PG1, PG6, PC4, and disappeared in model PC1. In the healthy cohort, unstable nodes were observed in a relatively larger number of models (39 out of 46) with respect to the other two cohorts. Consequently, statistically significant differences in the occurrence of unstable nodes emerged between the healthy cohort and both pre-CEA and post-CEA cohorts (Figure S1).

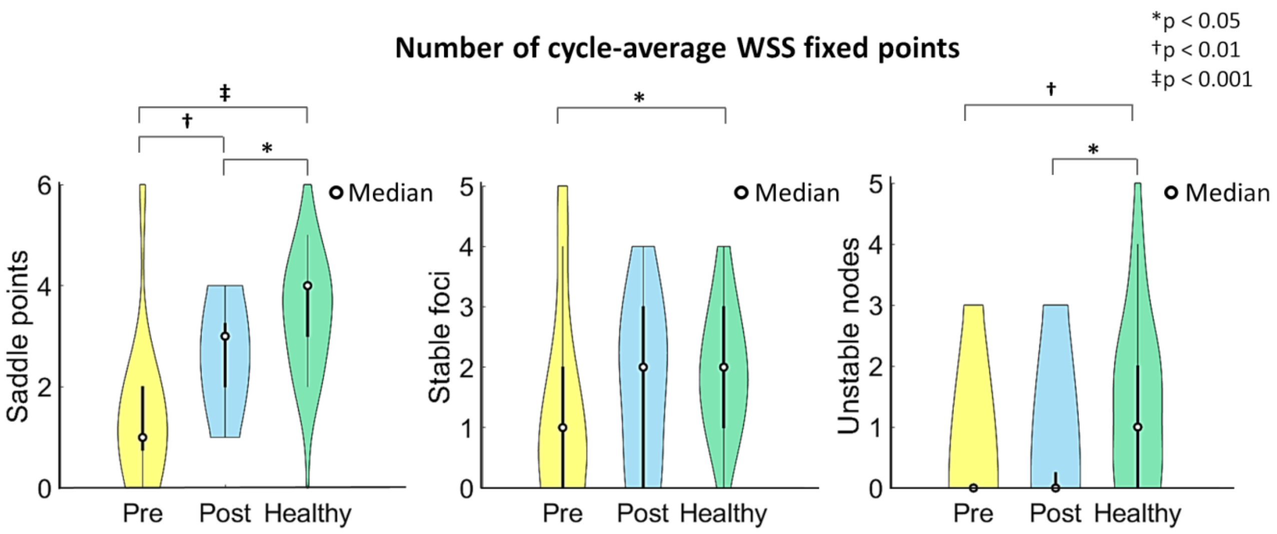

FIGURE S1. Violin plots of the occurrence of cycle-average WSS fixed points in the bifurcation region (delimited by sections CCA3-ICA5-ECA2) for pre-CEA, post-CEA and healthy cohorts. Distribution, median and quartile range are displayed for each cohort. Differences among the three cohorts are evaluated with a Wilcoxon signed-rank test.

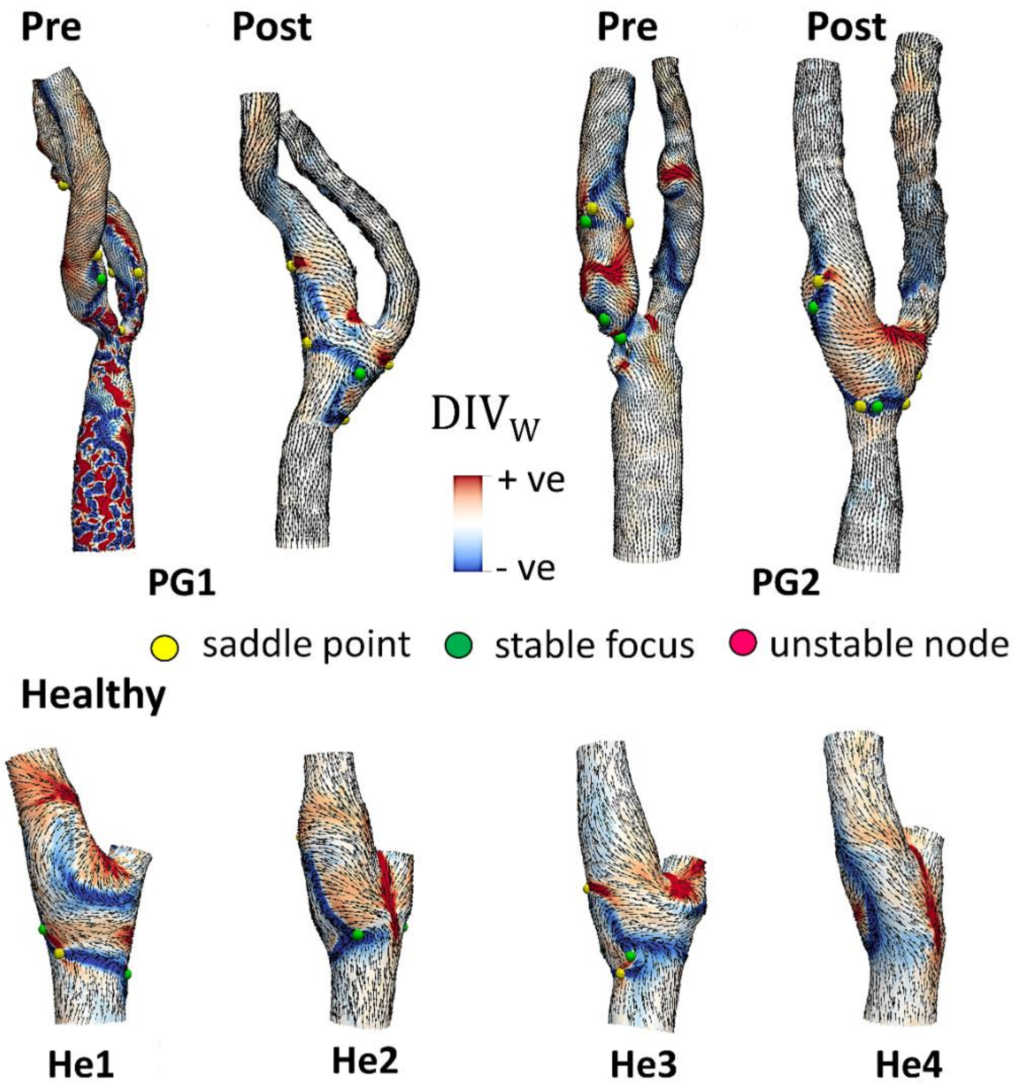

FIGURE S2. Topological skeleton of cycle-average WSS vector in selected pre-CEA (Pre), post-CEA (Post) and healthy cases. The topological skeleton in pre-CEA and post-CEA cohorts is extended beyond the bifurcation region (delimited by sections CCA3-ICA5-ECA2) to include in the pre-CEA models possible distal stenoses. Blue and red color define contraction and expansion regions, respectively, as given by the divergence of the normalized WSS vector field $\mathrm{DIV_W}$. The WSS vector field is normalized for visualization.

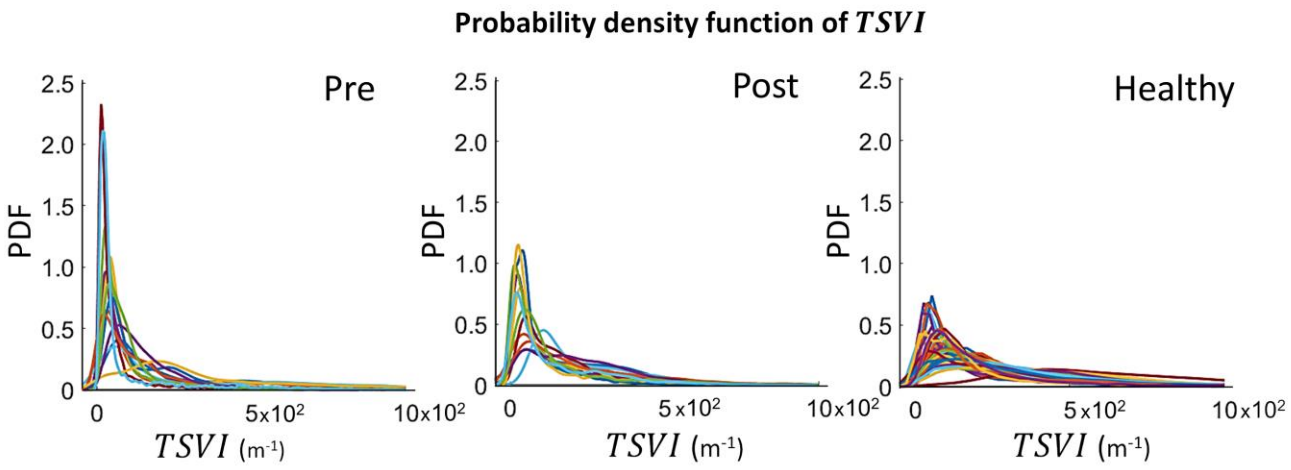

FIGURE S3. Probability density function of Topological Shear Variation Index (*TSVI*) in the bifurcation region (delimited by sections CCA3-ICA5-ECA2) for pre-CEA, post-CEA and healthy cohorts. Low *TSVI* values are associated with the highest probability for all three cohorts (right skewed distributions).

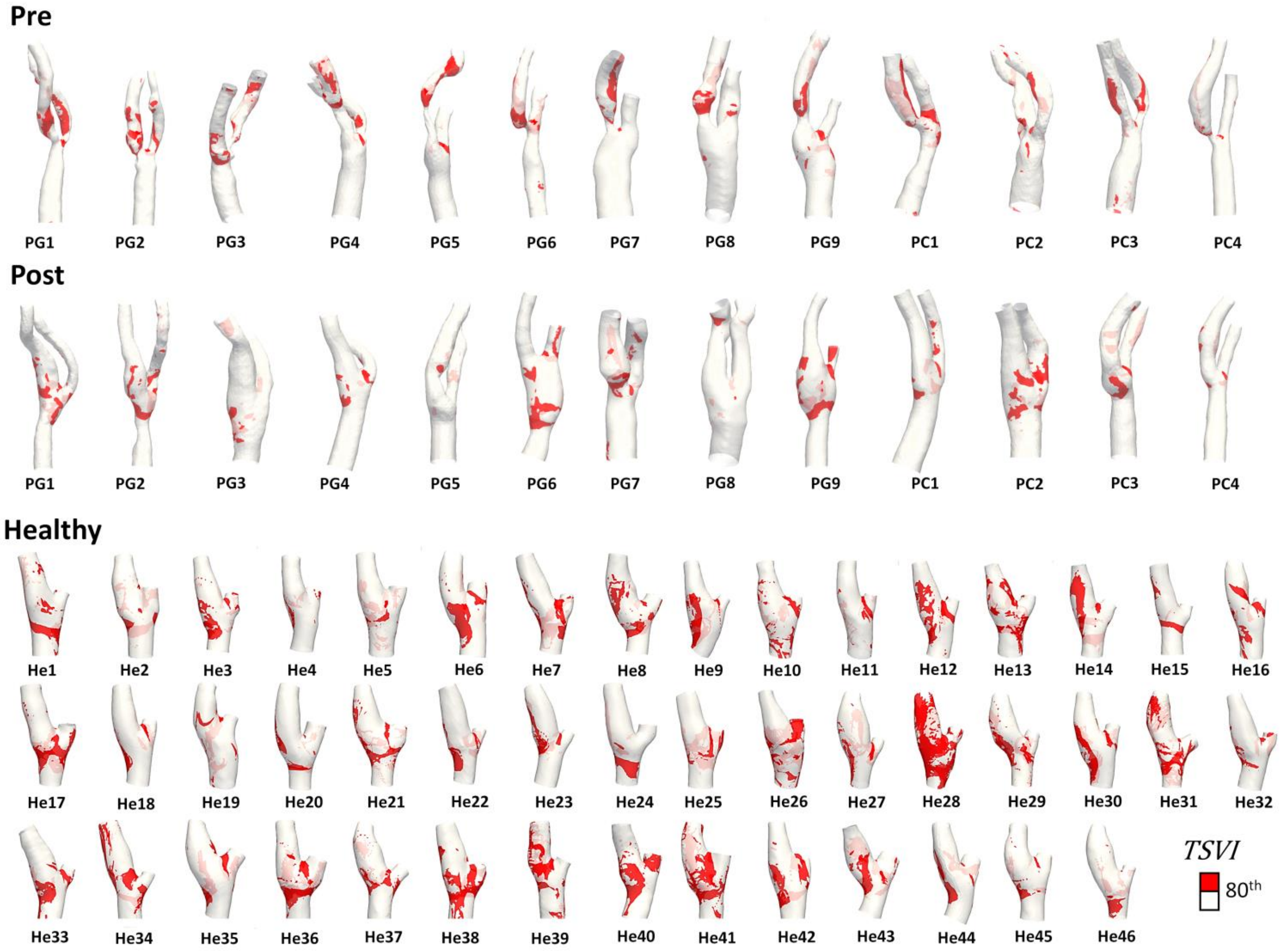

FIGURE S4. Luminal surface area exposed to high Topological Shear Variation Index ($TSVI$) value, as expressed by Topological Shear Variation Area (TSVA), in pre-CEA, post-CEA and healthy cohorts. The TSVA in pre-CEA and post-CEA cohorts is extended beyond the bifurcation region (delimited by sections CCA3-ICA5-ECA2) to include in the pre-CEA models possible distal stenoses. Red areas represent a $TSVI$ value above the 80th percentile of the pooled $TSVI$ distribution of the healthy models in the bifurcation region.

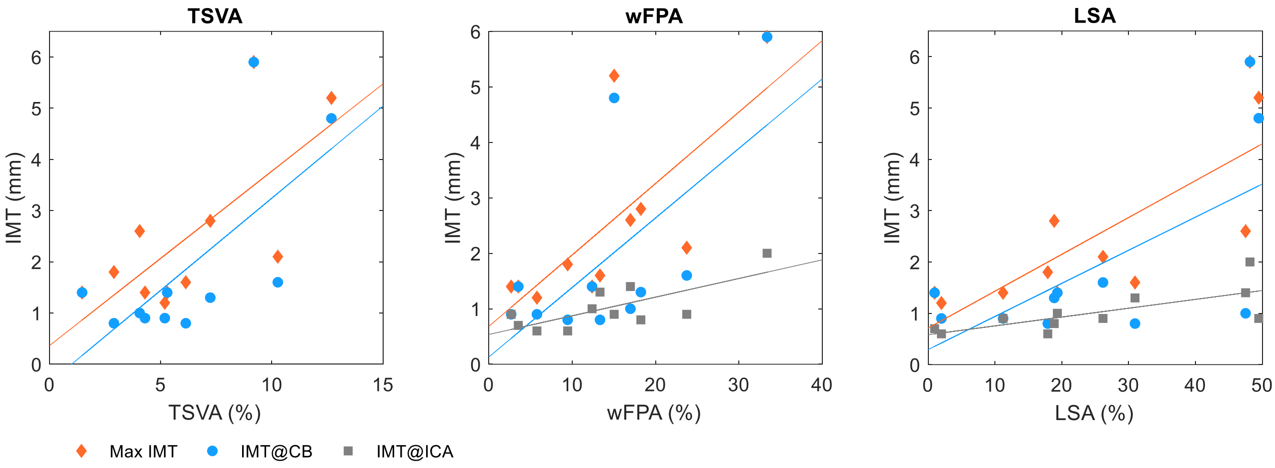

FIGURE S5. Scatter plots illustrating the significant associations between Topological Shear Variation Area (TSVA), weighted Fixed Points Area (wFPA) or Low Shear Area (LSA) vs. intima-media thickness (IMT) at 60 months follow up. CB: carotid bulb; ICA: internal carotid artery.

Table S1. Relationship between the hemodynamic variables weighted Fixed Points Area (wFPA), the type-specific wFPA (wFPA considering nodes: $wFPA_n$; wFPA considering focus: $wFPA_f$; wFPA considering saddle points: $wFPA_s$), and intima-media thickness (IMT) measurements.

**Coefficient R^2**	wFPA	$wFPA_n$	$wFPA_f$	$wFPA_s$
Maximum IMT	0.534*	0.235	0.505*	0.547†
IMT @ FD-2cm	0.108	0.390*	0.082	0.072
IMT @ FD-1cm	0.271	0.147	0.181	0.314
IMT @ FD	0.161	0.025	0.077	0.231
IMT @ CB	0.425*	0.212	0.425*	0.419*
IMT @ ICA	0.541†	0.308	0.506*	0.557†

* $p<0.05$; † $p<0.01$

IMT Intima-media thickness, CCA common carotid artery, CB carotid bulb, FD flow divider, ICA internal carotid artery. Maximum IMT, IMT measured at the bifurcation level (flow divider FD), CCA at 2cm and 1cm proximal to the FD (FD-2cm and FD-1cm), at the CB, at the ICA downstream of the CB.
